# Isolation and light chain shuffling of a *Plasmodium falciparum* AMA1-specific human monoclonal antibody with growth inhibitory activity

**DOI:** 10.1186/s12936-020-03548-3

**Published:** 2021-01-11

**Authors:** Melanie Seidel-Greven, Otchere Addai-Mensah, Holger Spiegel, Gwladys Nina Chiegoua Dipah, Stefan Schmitz, Gudrun Breuer, Margaret Frempong, Andreas Reimann, Torsten Klockenbring, Rainer Fischer, Stefan Barth, Rolf Fendel

**Affiliations:** 1 Fraunhofer Institute for Molecular Biology and Applied Ecology IME, Forckenbeckstr.6, 52074 Aachen, Germany; 2 Department of Medical Diagnostics, Faculty of Allied Health Sciences, Kwame Nkrumah University of Science and Technology, Kumasi, Ghana; 3 Department of Molecular Medicine, School of Medicine and Dentistry, Kwame Nkrumah University of Science and Technology, Kumasi, Ghana; 4 Institute of Molecular Biotechnology (Biology VII), RWTH Aachen University, Worringerweg 1, 52074 Aachen, Germany; 5 Purdue University, West Lafayette, IN 47907 USA; 6 Department of Experimental Medicine and Immunotherapy, Institute of Applied Medical Engineering, RWTH Aachen University Clinic, Pauwelsstraße 20, 52074 Aachen, Germany; 7 South African Research Chair in Cancer Biotechnology, Department of Integrative Biomedical Sciences, and Medical Biotechnology & Immunotherapy Research Unit, Institute of Infectious Disease & Molecular Medicine, Faculty of Health Sciences, University of Cape Town, Cape Town, South Africa; 8 Institute of Tropical Medicine, University of Tübingen, Wilhelmstraße 27, 72074 Tübingen, Germany

**Keywords:** Malaria, *Plasmodium falciparum*, Apical membrane antigen 1, Human monoclonal antibodies, Parasite growth inhibition, Phage display, Chain shuffling, Plant expression

## Abstract

**Background:**

*Plasmodium falciparum*, the parasite causing malaria, affects populations in many endemic countries threatening mainly individuals with low malaria immunity, especially children. Despite the approval of the first malaria vaccine Mosquirix™ and very promising data using cryopreserved *P. falciparum* sporozoites (PfSPZ), further research is needed to elucidate the mechanisms of humoral immunity for the development of next-generation vaccines and alternative malaria therapies including antibody therapy. A high prevalence of antibodies against AMA1 in immune individuals has made this antigen one of the major blood-stage vaccine candidates.

**Material and methods:**

Using antibody phage display, an AMA1-specific growth inhibitory human monoclonal antibody from a malaria-immune Fab library using a set of three AMA1 diversity covering variants (DiCo 1–3), which represents a wide range of AMA1 antigen sequences, was selected. The functionality of the selected clone was tested in vitro using a growth inhibition assay with *P. falciparum* strain 3D7. To potentially improve affinity and functional activity of the isolated antibody, a phage display mediated light chain shuffling was employed. The parental light chain was replaced with a light chain repertoire derived from the same population of human V genes, these selected antibodies were tested in binding tests and in functionality assays.

**Results:**

The selected parental antibody achieved a 50% effective concentration (EC_50_) of 1.25 mg/mL. The subsequent light chain shuffling led to the generation of four derivatives of the parental clone with higher expression levels, similar or increased affinity and improved EC_50_ against 3D7 of 0.29 mg/mL. Pairwise epitope mapping gave evidence for binding to AMA1 domain II without competing with RON2.

**Conclusion:**

We have thus shown that a compact immune human phage display library is sufficient for the isolation of potent inhibitory monoclonal antibodies and that minor sequence mutations dramatically increase expression levels in *Nicotiana benthamiana*. Interestingly, the antibody blocks parasite inhibition independently of binding to RON2, thus having a yet undescribed mode of action.

## Background

The malaria pathogen *Plasmodium falciparum* poses a threat to the populations of many of the world’s poorest countries as well as travelers from non-endemic areas. It causes 200 million infections per year, 500,000 deaths (mainly young children and pregnant women) and significant economic damage [1]. Strains of the parasite have emerged that show resistance towards previously efficient drugs, and only after many years of research has a first vaccine (Mosquirix™, GSK) been recently approved by the European Medicine Agency (EMA), but it still has to be evaluated in large-scale pilot implementation programmes [2].

The underlying mechanisms of acquired immunity to malaria are not completely understood. The resulting lack of knowledge regarding the role of innate, as well as B- and T-cell responses, complicates the development of an effective vaccine. However, the protection of patients by serum transfer from immune individuals was demonstrated as early as 1961 and is attributed to protective IgG antibodies most probably through blocking invasion of the pathogen into erythrocytes [3, 4]. Recently, sterile immunity was achieved in a clinical trial on malaria vaccine using chemo-attenuated fully viable cryopreserved Pf sporozoites (PfSPZ-CVac). The mode of action of this vaccine might be antibody-based blocking of hepatocyte invasion with the supplement of tissue residing T-cell memory response against the parasitic liver stage [5].

Malaria immunity is studied extensively by analysing the antibody reactivity and repertoire of individuals after vaccination, controlled human infection experiments or natural exposure and the sera of immunized rodents or simians. These provide useful insights for vaccine development and influence the selection of vaccine components [6–11]. One of the major vaccine candidates is apical membrane antigen 1 (AMA1), a blood stage antigen that is conserved among apicomplexan parasites and is required during erythrocyte invasion [12]. The antigen was proposed as a vaccine candidate based on the high prevalence of AMA1-antibodies in immune individuals [13–15]. Several studies have demonstrated that AMA1-specific immune IgG from animals immunized with recombinant AMA1 mediates efficient in vitro inhibition of parasite growth [16, 17]. The protective efficacy of AMA1 based vaccines has further been confirmed by animal challenge studies [18–20]. AMA1 has been tested in clinical trials and a positive correlation between protection against clinical malaria and the presence of AMA1-specific antibodies has been suggested because of the high prevalence of AMA1-antibodies in immune individuals [13–15]. AMA1 is initially synthesized as an 83 kDa protein, which is present on the merozoite surface as a 66 kDa derivative after proteolytic cleavage [21]. It plays an important role during erythrocyte invasion and is also found in the sporozoite stage of the parasite, where it plays a potential role in hepatocyte invasion [22].

The crystal structure of AMA1 reveals three domains. A conserved hydrophobic pocket surrounded by a cluster of polymorphic residues is found between domains I and II, which is necessary for binding of the rhoptry neck protein RON2 [23], a part of the macromolecular moving junction complex of proteins (RON2/4/5/8) secreted from parasite organelles known as rhoptries. RON2 interacts with AMA1 to form a moving junction between the merozoite and erythrocyte during erythrocyte invasion [24–26]. Invasion inhibitory antibodies block this interaction as one of their mechanisms of action [27]. Compared to other merozoite surface proteins AMA1 is highly polymorphic between different strains and isolates. Furthermore, it has been shown that allele-specific immune responses only provide good efficacy against homologous strains, while the inhibition of heterologous strains is poor [12, 28]. In vaccine development, two strategies have been followed to address the problem of AMA1 polymorphism: First, the immunization with recombinant protein mixtures derived from up to five different alleles [29, 30], and second, the design of three so called diversity covering (DiCo) variants that covered a large number of allelic variations within the context of artificial consensus molecules [31].

Several growth-inhibitory antibodies directed against *P. falciparum* AMA1 have been described, including murine mAb 1F9 and rat-mAb 4G2, which have been used to determine the mechanisms of erythrocyte invasion and antibody mediated protection [32]. In addition to their important function in the investigation of molecular and immunological mechanisms of host-parasite interactions, antibodies have been proposed as a treatment for patients who were non-responsive to standard therapy, as well as for non-immune travellers [33]. Approximately 79 monoclonal antibodies have been approved or are under review for the treatment of various diseases, including infectious diseases such as infectious diseases of RSV and Clostridium difficile [34, 35]. Because of their stoichiometric mechanism of action, many therapeutic antibodies have to be applied in large quantities (several hundred mg per dose) which increases the treatment costs per patient [36–38]. This makes current fermentation-based production strategies unsuitable for a disease like malaria, which disproportionately affects people from the world’s poorest regions [39]. Furthermore they should have a low immunogenicity, achieved either by the humanization of animal antibodies or the isolation of human antibodies [40]. Phage display permits the selection of human antibodies to virtually any antigen, when a library is created from the variable immunoglobulin region genes isolated from human B cells [41]. In fact, the first human therapeutic monoclonal antibody Adalimumab (Humira) was generated by phage display. Two more phage display derived human monoclonal antibodies (AbThrax, Benlysta) are on the market and more than 20 are in clinical trials [42]. Moreover, when human monoclonal antibodies are isolated from immune antibody libraries, functional and genetic analyses may contribute to the investigation of mechanisms of the human immune system, although the in vivo pairing of heavy and light chain is not guaranteed as the cloning procedure creates random pairs of heavy and light chains. Another feature of this method is that it can be used for affinity maturation of antibodies [43]. Thus, phage display is not only helpful for the development and optimization of therapeutic human antibodies but is also a suitable method for the investigation of naturally occurring human anti-malarial antibodies, contributing to the elucidation of the complex mechanism of malaria immunity, which in turn impacts vaccine design.

Here the construction of an immune phage display Fab library from the variable region genes of semi- immune donors from an endemic region in Ghana and selected AMA1-specific antibodies by four rounds of solid phase panning against a mixture of diversity covering variants of AMA1 is described. After initial characterization, a light chain shuffling approach is used for possible affinity maturation of an isolated antibody and identified four combinations with four different light chains. The antibodies were transiently expressed in *Nicotiana benthamiana* plants and characterized with respect to sequence, allele specific affinity and strain specific functional activity.

## Methods

### Bacterial strains, phage strains, parasite strains and antigens

*Escherichia coli* XL1-Blue MRFʹ was purchased from Agilent Technologies. *Escherichia coli* Dh5α and HB2151 cells were purchased from Nordic Bio site. *Agrobacterium tumefaciens* strain GV3101: pMP90RK [GmR, KmR, RifR] [44] and *N. benthamiana* plants were used for the production of recombinant proteins by agroinfiltration. Phagemid pRFII is a derivative of pHen1 adapted for Fab-expression [45]. Phagemid pAK400 was kindly provided by Andreas Plückthun (University of Zürich, Switzerland). *Plasmodium falciparum* strains 3D7, K1, HB3, W2mef, and FCR3 were obtained through the MR4 as part of the BEI Resources Repository, NIAID, NIH. Recombinant AMA1 variants DiCo1, 2 and 3 were kindly provided by Ed Remarque (BPRC Rijswijk, The Netherlands) [31]. Recombinant koAMA1 (3D7 strain) was kindly provided by Alexander Boes (Fraunhofer IME, Aachen) [17].

### Blood donor population

The blood donors (ages 25–45) were from malaria endemic region Kumasi, Ghana. The major selection criterion was supposed malaria immunity indicated by the self-reported absence of clinical malaria and malaria treatment for a minimum of 2 years. Pregnant and nursing women, as well as individuals with known disease conditions, inflammatory conditions or anaemia, were excluded. Based on malaria-specific antibody levels previously determined [46], 50 blood donors were selected for the generation of the Fab phage display library.

### Ethical approval

Written informed consent was obtained from all participants after the goals of the project had been thoroughly explained to them. Ethical approval for the study was obtained from the Committee on Human Research Publication and Ethics (CHRPE) of the Kwame Nkrumah University of Science and Technology in Kumasi, Ghana.

### RNA isolation and cDNA synthesis

Blood was collected from 50 selected donors in PAXgene blood RNA tubes (PAXgene Blood RNA Kit, PreAnalytiX, 762165) at the phlebotomy site in Kumasi, incubated at room temperature for 2 h and stored at − 80 °C for transport. Blood samples in PAXgene blood RNA tubes were thawed on ice, incubated at room temperature for 2 h and total RNA was extracted according to the manufacturer’s instructions.

cDNA was prepared separately from each sample by reverse transcription of 8 µL of total RNA using the Invitrogen Superscript III First strand synthesis system for RT-PCR (18080-051) and oligo-dT primers according to the manufacturer’s instructions.

### V gene amplification and Fab library construction

V genes were amplified from 2 µL of pooled cDNA sourced from 50 donors, using a human V gene primer set (see Additional file 1: Table S1). Each reaction comprised 0.2 mM dNTPs, 1.5 mM MgCl_2_ and 0.2 µM of one forward and the reverse primer, and 2 units of Platinum Taq DNA polymerase (Invitrogen, 10966-018). The sample was denatured at 95 °C for 10 min followed by 30 cycles of denaturation at 95 °C for 1 min, annealing at 55 °C (kappa) and 63 °C (heavy) for 1 min, and elongation 72 °C for 2 min, and a final elongation step for 10 min at 72 °C. The PCR products were re-amplified to add the restriction sites SalI and SfiI (heavy chain), and ApaLI and NotI (κ light chain).

The PCR products from all primer combinations were mixed for the transfer to the phagemid pRFII. The immune Fab library was constructed in pRFII by two-step cloning, starting with the kappa light chains. Insert and vector sequences were double digested with the restriction endonucleases ApaLI (R0507) and NotI (R0189) and ligated with T4 DNA ligase (M0202S) purchased from NEB. A quantity of 200–500 ng of the ligation products were introduced into 25 µL of thawed *E. coli* XL1blue MRFʹ cells in 0.1-cm gap electroporation cuvettes (Bio-Rad, 165-2089). The light chain library was isolated from pooled colonies with the NucleoBond® Xtra Midi/Maxi kit (Macherey Nagel, 740414.50) according to the manufacturer’s instructions. The light chain library and heavy chain inserts were digested with SfiI (NEB, R0123) and SalI-HF® (NEB, R3138) and cloned as described above for the light chain library.

### Light chain shuffling library construction

For the construction of a light chain shuffling library, plasmid DNA was prepared using the NucleoSpin® Plasmid kit (Macherey Nagel, 740588.50). The vectors were digested with ApaLI and NotI, and the light chain repertoire was amplified and cloned as described above. For subcloning into pFlx the final light chain library was transferred from pRFII using a NotI and a C-terminal SfiI site.

### Nucleotide sequence analysis

For quality assessment of the libraries and sequence analysis of selected binders as well as control of cloned constructs, it was sequenced with an ABI PRISM® 3730 Genetic Analyzer (Applied Biosystems, Carlsbad, USA). Sequence analysis was done using CLC Main Workbench and the IMGT/V-quest online tool [47].

### Phage display biopanning

Phage libraries were rescued by infection with 1 × 10^10^ pfu of M13KO7 helper phage (NEB, 0315S) or M13K7ΔpIII hyper phage (Progen, PRHYPE) per mL of bacteria at OD_600nm_ 0.5 (moi 20:1), followed by overnight amplification at 30 °C in 2xTY supplemented with 100 μg/mL ampicillin (pRF) or chloramphenicol (pFlx) and 30 μg/mL kanamycin. The phage containing culture supernatant was harvested by centrifugation (4000×*g*, 4 °C, 40 min). Phages were precipitated by incubating the supernatant with 20% polyethylengycol (PEG 6000) and 2.5 M NaCl on ice for 1 h followed by ultra-centrifugation (12,000×*g*, 4 °C, 40 min). The phages were resuspended in PBS, cell debris was removed by centrifugation (16,000×*g*, 4 °C, 5 min) and passing through a 0.2-µm syringe filter, and the phages were then used for bio-panning.

Four rounds of selection were carried out with decreasing antigen concentrations as follows: 10 μg/well for round 1, 5 μg/well for round 2, and 1 μg/well for rounds 3 and 4.

A mixture containing equal amounts of the three diversity-covering AMA1 DiCo1–3 in phosphate buffered saline (PBS) was coated overnight at 4 °C onto 96-well high binding plates (Greiner Bio-One). The next day, the wells were rinsed with PBS containing 0.1% (v/v) Tween-20 (PBST) and blocked with 2% (w/v) skimmed milk powder in PBST (MPBST) for 1 h at room temperature. Phage particles were blocked in 2% (w/v) MPBST for 1 h at room temperature and then depleted in similarly blocked wells for 2 h at room temperature. The unbound phages were transferred from the depletion wells to the antigen-coated wells and incubated for a further 1 h at room temperature. Unbound unspecific phages were removed by increasing the washing stringency (10, 20, 30, 40, washes with PBST) in every panning round. Bound phages were eluted in 200 µL/well of 200 mM glycine at pH 2.2 and in parallel with 100 mM triethanolamine for 10 min at room temperature and immediately neutralized with 1 M Tris–HCl (pH 9 and pH 7.4, respectively). Input and output phages were used to infect log-phase *E. coli* XL1-Blue cells (OD_600nm_ = 0.5) and were plated onto 2xTY agar plates supplemented with 100 μg/mL ampicillin and 2–4% (w/v) glucose at 37 °C. Colonies were scraped off into 2xTY containing 30% (v/v) glycerol and the appropriate antibiotics and stored at − 80 °C or immediately inoculated at OD_600nm_ < 0.1 into 2xTY + ampicillin or chloramphenicol for a further round of panning. Titration plates were used to calculate the input and output titers.

### Monoclonal phage ELISA

Single colonies from the last panning round were inoculated into 200 µL 2xTY medium supplemented with ampicillin and glucose in 96-well round-bottomed culture plates and were grown overnight at 37 °C shaking at 200 rpm. A volume of 10 µL of these pre-cultures were transferred to another 200 µL 2xTY medium and incubated for 3 h until early log phase and infected with 2 × 10^7^ pfu of M13KO7 helper phages. The infected bacteria were pelleted, and the culture medium replaced with glucose-free 2xTY supplemented with kanamycin, and ampicillin (pRFII) or chloramphenicol (pFlx) and the phages were amplified at 30 °C overnight shaking at 200 rpm.

The phage-containing supernatant was harvested by centrifugation (1300×*g*, 4 °C, 10 min) and a high-binding microtiter plate was coated with between 100 and 500 ng of antigen per well overnight at 4 °C. After rinsing three times with PBST 100 µL of the phage supernatant was incubated for 1 h at room temperature on the antigen. Unbound phage-Fab particles were washed away with three PBST washes, and mouse monoclonal anti-M13/fd/F1, B62-FE2, (Progen, 61097, diluted 1:500 in PBS) was added and incubated for 1 h. After three further washes with PBST, a goat-anti-mouse detection antibody conjugated with alkaline phosphatase (Dianova, 115055-033) or horseradish peroxidase (Sigma, A2554) was added at a dilution of 1:5000 in PBS and incubated for 1 h at room temperature. The plates were washed five times with PBST before adding substrate solution [pNPP (Sigma-Aldrich, S0942) or TMB (Thermo Fisher Scientific, 002023)] for 30–60 min or until the signal was clear. Positive clones were identified by measuring the absorbance at 450 or 650 nm and defined as clones with an OD value 5–10 times higher than the background signal (PBS).

### Cloning, expression and purification of IgGs

Variable genes of positive binders from the phage ELISA (or soluble Fab ELISA) were amplified with cloning V,J-primers (see Additional file 1: Table S2) featuring restriction sites AgeI and BsiWI (kappa) or SalI (heavy) and cloned into a modified version of the plant expression vector pTRAkt as previously described [48, 49]. Ligations were performed with T4DNA ligase (NEB, M0202S) according to the manufacturer’s instructions and introduced into chemically-competent *E. coli* DH5α cells. Positive clones were identified by colony PCR and sequencing. The subsequent generation and cultivation of recombinant *A. tumefaciens* (strain GV3101: pMP90RK) as well as the transient expression of antibody variants in *N. benthamiana* plants (4–6 weeks old) was performed as previously described [50].

Leaves were harvested and homogenized in a blender using two volumes of ice-cold PBS supplemented with 10 mM sodium metabisulfite. The homogenate was centrifuged (38,400×*g*, 4 °C, 40 min) and filtered through a double layer of Miracloth (Merck Millipore, 475855-1R) to remove leaf debris. The supernatant was mixed with 0.5 M sodium chloride, adjusted to pH 8 and incubated on ice for 30 min. The precipitated plant proteins were removed by centrifugation as described above and the supernatant was passed through a 0.2-µm filter.

Immunoglobulins were purified by protein A affinity chromatography using Protein A Ceramic HyperD® F sorbent [51]. Antibodies were eluted with 0.2 M sodium citrate, pH 2.5, and elution fractions neutralized with 1 M Tris–HCl (pH 8) and dialyzed against PBS. For growth inhibition assays, antibodies were concentrated using Hydrosart® Vivaspin® 15R Centrifugal Concentrators (30,000 MWCO, Sartorius, VS15RH21), buffer exchanged against RPMI and passed through a sterile filter (Ø 0.2 µm) and stored at − 20 °C.

### Indirect ELISA

Indirect ELISA was used for the determination of specificity of full size human monoclonal antibodies. All incubation steps were performed in the wells of a 96 well plate at a 100 µL scale at room temperature for 1 h or overnight at 4 °C. It was washed with PBST three times after every incubation step. Before the addition of substrate it was washed 5 times. As a first step, antigens were diluted in PBS at concentrations of 500 ng/mL to 2 ng/mL and immobilized on the plate by incubation at 4 °C overnight or room temperature for 2 h. Secondly, it was blocked with blocking buffers MPBS or Roti®-block (Carl Roth). As a third incubation step, the primary antibody was added either as purified antibody diluted in PBS or blocking buffer. Subsequently anti-human-IgG/H+ )-AP (Promega, S3821) or anti-human-IgG (H+L)-HRP (Promega, W4031) was added at a dilution of 1:5000 in PBS or in blocking buffer. Finally, 100 µL/well substrate pNPP or TMB was added. For pNPP the absorption at 405 nm was measured after 30–60 min substrate incubation. The TMB reaction was stopped with 100 µL/well 1 M HCl and the absorbance was measured at 450 nm.

### Affinity and competition studies by surface plasmon resonance

Antibody–antigen binding was analysed by SPR spectroscopy using a Biacore T200 instrument (Biacore, GE Healthcare) and CM5-S-Series sensor chips with recombinant Protein A prepared as described previously [52]. All measurements were performed at 25 °C using HBS-EP (10 mM (4-(2-hydroxyethyl)-1-piperazineethanesulfonic acid), 150 mM NaCl, 3 mM EDTA, 0.005% (w/v) polysorbat-20) as the running buffer. For kinetic analysis, 100–150 RU of the different antibodies were captured onto immobilized protein A, and the recombinant AMA1 variants were injected at a flow rate of 30 µL/min for 180 s. Dissociation was followed for 900 s. Between measurements the surface was regenerated by pulsing for 1 min with 30 mM HCl. Buffer injections were used for double referencing. Binding curves were evaluated using Biacore T200 Evaluation Software (GE Healthcare).

For competition experiments, the AMA1 antibodies were immobilized on a sensor chip. AMA1 was captured and two of the anti-AMA1 antibodies were injected consecutively to analyse if binding was impaired. Clone 1D7 (MRA-480) was obtained from BEI Resources. To investigate if the antibody-AMA1 interaction is blocked or weakened by the binding of PfRON2sp1 (a peptide derived from natural AMA1 ligand RON2), the antibodies were captured on a protein A chip and AMA1 (DiCo3) alone or AMA1 (DiCo3), preincubated with a saturating µM concentration of PfRON2sp1, were injected and compared.

### Immunofluorescence microscopy

Synchronous *P. falciparum* 3D7A schizonts were mounted onto glass slides and fixed in methanol for 10 min at − 20 °C, then blocked using 1% (v/v) FCS in PBS for 1 h at room temperature and rinsed with PBS. Antibodies were diluted in PBS containing 1% (v/v) FCS and 0.01% (w/v) saponin. Slides were incubated with 50 μg/mL mouse-anti-MSP4 antibody 2.44 (kindly provided by Alexander Boes, Fraunhofer IME, Aachen) [49] in PBS containing 1% (v/v) FCS (IgG depleted) and 0.01% (w/v) saponin combined with 100 ng/mL of one of the human anti-AMA1 antibodies at room temperature for 1 h. An AMA1 specific human antibody 1E4 (Maskus et al., pers. commun.) and polyclonal anti-AMA1 antibody BG98 were used as positive control [53], whereas negative control slides were only stained with mouse-anti-MSP4 2.44. Slides were rinsed thoroughly with PBS and incubated with the detection antibodies [goat-anti-mouse IgG(H+L)-Alexa Fluor® 488 (Thermo Scientific, A-11001) diluted 1:100, or goat-anti-human IgG(H+L)-Cy3 (Dianova, 109–165-003) diluted 1:1000] at room temperature for 1 h. The slides were rinsed, allowed to dry in the dark and preserved using Prolong® Antifade mounting medium with DAPI (Thermo Scientific, P36935). Images were captured using a Leica TCS SP8 confocal microscope (Leica, Wetzlar, Germany).

### Parasite culture and synchronization

*Plasmodium falciparum* strains were cultured as previously described [54] in RPMI 1640, containing 25 mM HEPES, 2 mM l-glutamine, 50 μg/mL gentamycin, 10% (w/v) Albumax®II (Life Technologies) at a haematocrit of 5%. (v/v) Erythrocytes (group O Rh^+^) were obtained in CPDA tubes from the Department for Transfusion Medicine, RWTH Aachen University Clinic, Aachen, Germany. The erythrocytes were washed three times in RPMI, centrifuged (600×*g*, room temperature, 5 min) and resuspended in SAG-M (150 mM NaCl, 50 mM d-glucose, 1.2 mM adenine, 28.8 mM d(−)-mannitol) at a haematocrit of 66.6%. (v/v) *P. falciparum* strains were synchronized by three sorbitol treatments 5, 3 and 1 days before the growth inhibition assays, as described elsewhere [55].

### In vitro growth inhibition assays

Growth inhibition assays were carried out as previously described with synchronized *P. falciparum* schizonts [50]. Growth inhibitory activity was measured in triplicate using antibodies diluted in RPMI 1640 containing 25 mM HEPES. Naïve polyclonal rabbit IgG (6 mg/mL) was used as a negative control and was measured in duplicate. Polyclonal rabbit-anti-AMA1 IgG (BG98) or human anti-AMA1 antibody 1E4 was used as a positive control, and was also measured in duplicate. The plant produced human anti-HIV antibody 2G12 produced in plants was used as a further negative control [56, 57].

Schizonts were diluted at 0.3% parasitemia and 2% (v/v) hematocrit into 50 µL medium per well in a 96-well flat-bottomed half-area culture plate. They were harvested after approx. 48 h, washed with PBS and stored at − 80 °C. Parasitemia was determined by pLDH-assay. The percentage of relative growth inhibition was calculated as % inhibition = 100 × [A655 nm (sample) − A655 nm (erythrocyte control)]/[A655 nm (schizont growth control) − A655 nm (erythrocyte control)].

## Results

### Construction of a diverse immune Fab library

In a previous work, a set of 78 adult Ghanaian blood donors, and subsequently in a second blood withdrawal a subset of 31 Ghanaian blood donors, were screened by ELISA for IgG reactivity against several antigens, including AMA1: 71% and 97% of these volunteers (donors) showed a positive reaction against for AMA1, respectively [46, 58].

In order to generate a semi-immune Fab-derived library from 50 blood donors with highest ELISA reactivity, a two-step cloning strategy was used and the first κ-sub-library contained 7.6 × 10^6^ clones. Restriction of the pRFII-κ-sub-library was then followed by cloning the heavy-chain V genes, yielding a total library size of 1.9 × 10^7^ clones and a functional (net) size of 1.4 × 10^7^, as determined by colony PCR.

**Fig. 1:**
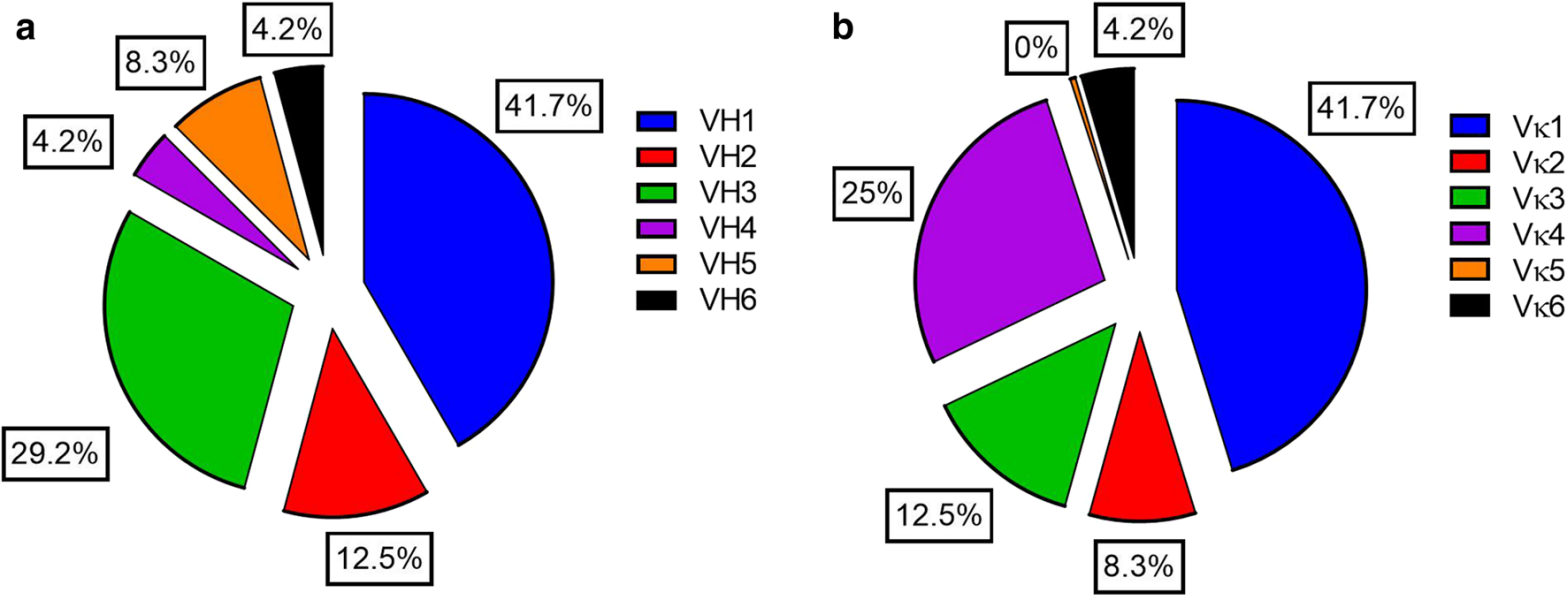
Percentages of VH (**a**) and Vκ families (**b**) within the Malaria-immune combinatorial Fab phage display library. The library was derived from the whole blood RNA (cDNA) of 50 Ghanaian malaria-immune donors and had a functional size of 1.4 × 10^7^ clones. Pie charts represent the percentages of V gene families calculated after sequence analysis of 24 random clones. V gene usage was analysed using IMGT/V-Quest

Then, 24 random clones were selected from the final library and analysed the sequence diversity and V gene usage. Heavy chain sequencing revealed that all six functional VH families were represented with a predominance of the VH1 family (Fig. 1a), whereas light chain sequencing revealed that five of the six Vκ families were represented (Fig. 1b), only Vκ family number 5 was not present in the analysed sample set. Sequence analysis also confirmed a high library diversity, with no redundancy among the analysed clones.

### Selection of AMA1-binders

Four successive rounds of panning on an equal mixture of AMA1 variants DiCo1–3 achieved a final 1250-fold enrichment with a progressive increase in the percentage of clones with inserts of the correct size from the first to the fourth selection round. Enrichment is defined as the output/input ratio in any panning round compared to the same ratio in the first panning round (Table 1).

**Table 1:** Panning of the Malaria immune combinatorial library

Selection round	Input titer	Output titer	Output/input	Enrichment		Clones with inserts	
				Per round	Total		
1	1.6 × 10^12^	1.9 × 10^6^	1.2 × 10^–6^	–	–	8.3%	(2/24)
2	1.2 × 10^12^	9.0 × 10^6^	7.5 × 10^–6^	6.25 ×	6.25 ×	36.8%	(7/19)
3	2.5 × 10^11^	3.0 × 10^6^	1.2 × 10^–5^	1.6 ×	10 ×	50%	(14/28)
4	2.0 × 10^11^	3.0 × 10^8^	1.5 × 10^–3^	125 ×	1250 ×	95.6%	(28/29)

The library was panned using helper phages M13KO7 in four rounds of panning with increasing washing stringency and decreasing antigen concentration. Input and output titers were titrated throughout four selection rounds. The enrichment from round to round and total enrichment were calculated. The percentage of clones with inserts of the correct size was determined by colony PCR

After each panning round, the positive clones were sequenced, and their closest germline gene matches identified using IMGT/V-Quest. After the fourth selection round, 29 individual representative clones were picked and sequenced. Out of these, 28 clones contained a full-length Fab sequence, and 22 (78.6%) contained one specific selected clone.

### Screening for specificity to AMA1 (DiCo1-3)

**Fig. 2:**
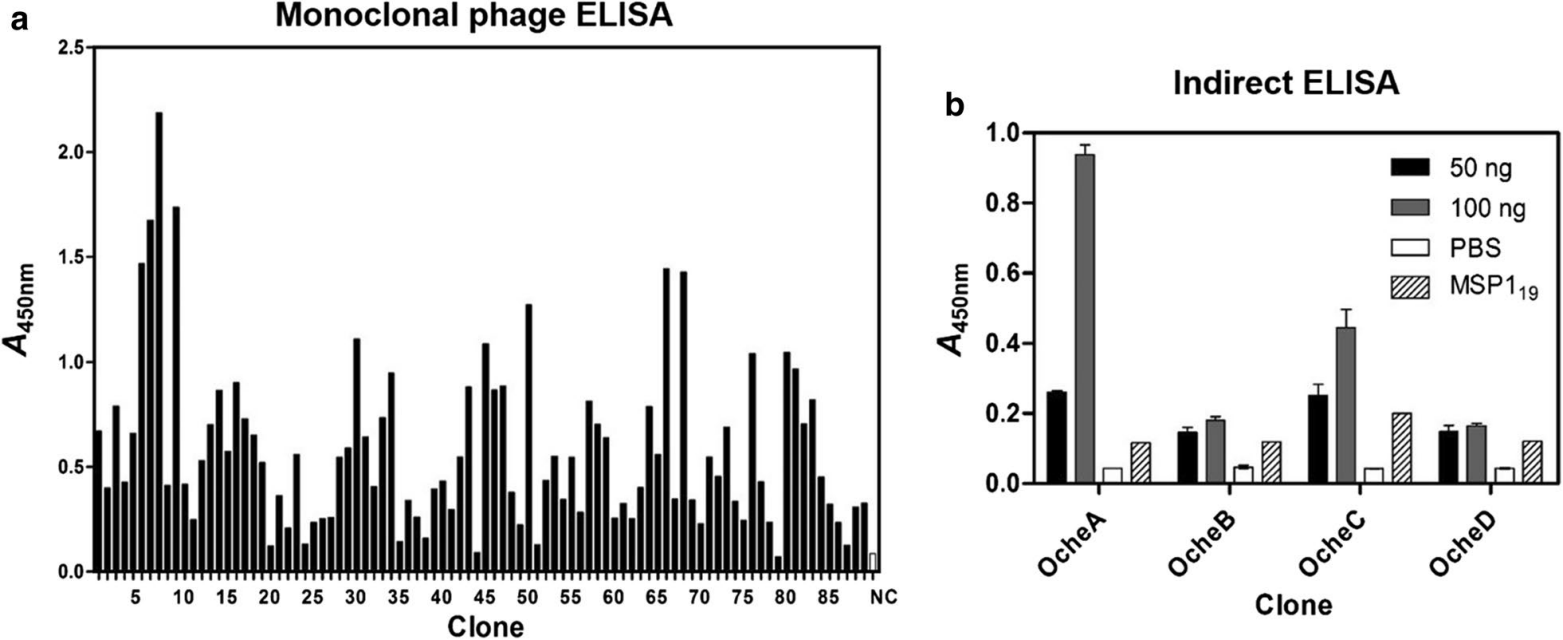
Monoclonal phage ELISA of phage display output clones of round 4 and indirect ELISA of full-size antibodies of phage ELISA positive clones. **a** 89 output clones of the last phage display panning round (round four) were tested in monoclonal phage ELISA against AMA1 DiCo1-3. Detection was performed using antiM13 antibody. As negative control, providing the background absorbance signal, the supernatant of uninfected XL1-Blue MRF’ was used. **b** The binding capacity of four selected full-size antibodies was estimated by indirect ELISA using shuffled variants of two heavy and two light chains of two antibodies that were positive for binding against DiCO1-3 in monoclonal phage ELISA. The antibodies were transiently expressed in *N. benthamiana*. As negative controls, the reaction against PBS and antigen MSP1_19_ was measured

A monoclonal phage ELISA against 96 randomly selected clones from selection round four identified six different binding clones. A clone representing each sequence was analysed by soluble Fab ELISA against 500 ng of antigen per well. Four clones were identified with a signal greater than twice the background signal. Two binders showed a signal at least 10 times higher than the background absorbance and were selected for further characterization (Fig. 2a).

After cloning the variable region genes of the two selected binders into the plant expression vector pTRAkt with appropriate primers, the original combinations and opposing shuffled combinations of the heavy and light chains were transiently expressed as full-size human IgG1/κ antibodies in *N. benthamiana* and were designated as clones “OcheA, B, C and D”. The expression levels of around 6 mg/kg fresh leaf weight (FLW) of tobacco plants were comparably low [49, 50].

The specificity of the full-size antibodies in plant extracts was analysed by ELISA using 50 ng and 100 ng of AMA1 DiCo1-3. OcheA showed the highest signal, followed by clone OcheC with the same heavy chain (Fig. 2b). Clones OcheB and OcheD (heavy chain from clone B) only showed a marginally increased signal compared to the negative controls (PBS and the antigen MSP1_19_). These results were confirmed by SPR spectroscopy with only clone OcheA showing significant binding to AMA1 DiCo3 with an affinity constant (K_D_) of ~ 1.4 × 10^–7^ M. The functionality of clone OcheA was tested in vitro using a growth inhibition assay. OcheA inhibited *P. falciparum* strain 3D7 ~ 64.5% at a concentration of 2.25 mg/mL and a 50%-effective concentration (EC_50_) of 1.25 mg/mL.

OcheA showed 88.19% (254/288 nt) germline identity to the Heavy V gene allele “Homsap IGHV1-69*11 F” and 83.33% (40/48 nt) identity to the J gene allele “Homsap IGHJ4*02 F” as analysed using IMGT/V-Quest. The heavy chain of OcheA showed a higher degree of maturation than the paired kappa light chain, matching the V gene allele “Homsap IGKV1-17*03 F” with 99.28% (277/279 nt) identity and the J gene alleles “Homsap IGKJ2*01 F and Homsap IGKJ2*02 F” with 92.11% (35/38 nt) identity.

To further improve the binding and inhibitory characteristics of this antibody, we proceeded with a light chain shuffling by phage display.

### Light chain shuffling

**Fig. 3:**
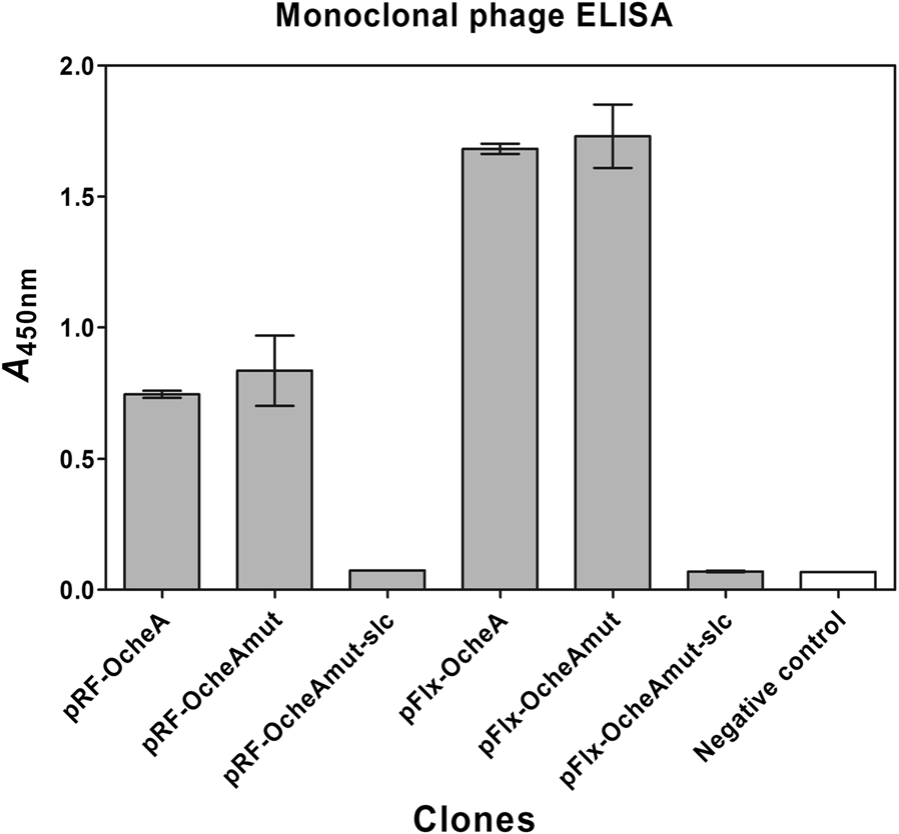
Monoclonal phage ELISA of OcheA constructs against AMA1 DiCo1-3. The ELISA was carried out with phage supernatants expressed from OcheA constructs in phagemids pRF or pFlx. Constructs containing the stuffer light chain (slc) did not bind to AMA1 DiCo1-3 as opposed to the complete Fab-phage particles. Negative control: Untransformed XL1 blue supernatant

For the light chain shuffling, it was necessary to introduce a silent mutation in the heavy chain constant region of antibody OcheA to remove an ApaLI restriction site, as another ApaLI site was subsequently used for the replacement of the parental kappa light chain. Subsequently, the light chain of OcheA was exchanged with a 380-bp stuffer fragment (pRFOcheAmut-slc) to abrogate the binding to AMA1 and to avoid isolating the parental light chain from the light chain library as religations after incomplete restriction of the vector. The successful elimination of background binding activity was confirmed by phage ELISA (Fig. 3). Consecutively, the stuffer fragment was replaced with a human kappa chain repertoire with a final library size of 1.5 × 10^6^ clones as determined by titration. The quality of the library in terms of insert percentage and diversity was determined by colony PCR and sequencing. Sufficient quality was confirmed with an insert rate of ~ 80% and with all 26 sequenced clones having a different sequence.

The light chain repertoire was subcloned into the phagemid pFlx for comparative panning in a different phagemid system. To cover an approximate diversity as in the vector pRFII, the sub-library was extended to a size of 1.5 × 10^7^ clones, corresponding to ten times the size of the pRFII library. The sub-library had over 95% clones with inserts of the correct size and a high diversity confirmed by the sequencing of 38 clones. OcheAmut-slc was subcloned into the vector pFlx. In a monoclonal phage ELISA, the inability of the OcheAmut-slc constructs (in pRF and pFlx) to bind to the AMA1 DiCo1-3 mix was confirmed as demonstrated by absorptions in the range of the negative control (uninfected *E. coli* XL1-Blue).

### Selection of clones using light chain shuffled phage display library

**Fig. 4:**
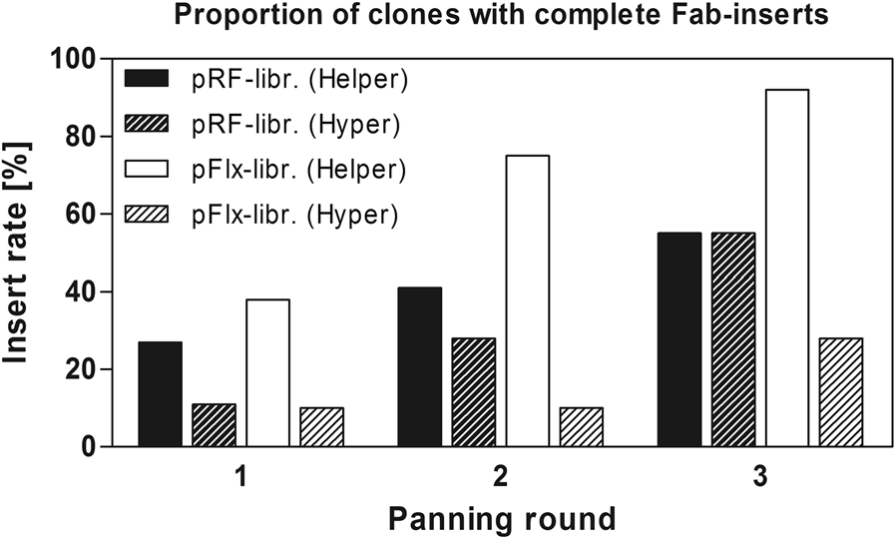
Proportion of output clones with complete Fab inserts as determined by colony PCR. The percentage of clones with complete Fab inserts was assessed after every panning round by analysing 20 clones per round via colony PCR with primers flanking the Fab insert

For the selection of antibodies, both libraries were panned in three successive rounds using helper phage MK13KO7 for packaging or hyper phage MK13KO7ΔpIII as an alternative approach. An increase in output titers as well as an enrichment of clones was observed for all setups (Table 2). The final yield of clones with inserts of the correct size in round 3 was highest for the pFlx library packaged using helper phages (Fig. 4).

**Table 2:** Light chain shuffling results

Panning round	Washing repeats	Antigen amount (µg/well)	Output/ input phage titer (total enrichment)			
			pRF-library		pFlx-library	
			Helper	Hyper	Helper	Hyper
1	10	10	1.6 × 10^–6^	4.1 × 10^–6^	9.8 × 10^–6^	4.7 × 10^–7^
2	25	5	1.2 × 10^–5^ (7.5 ×)	2.3 × 10^–6^ (0.6 ×)	1.4 × 10^–5^ (1.4 ×)	1.4 × 10^–6^ (3 ×)
3	40	1	2.9 × 10^–3^ (1812.5 ×)	3.6 × 10^–6^ (0.9 ×)	3.5 × 10^–4^ (35.7 ×)	8.8 × 10^–4^ (1872 ×)

The output and input titers were titrated for every selection round. The enrichment was calculated by dividing the output/input titers and setting the output/input ratio of round 1 as 1

**Fig. 5:**
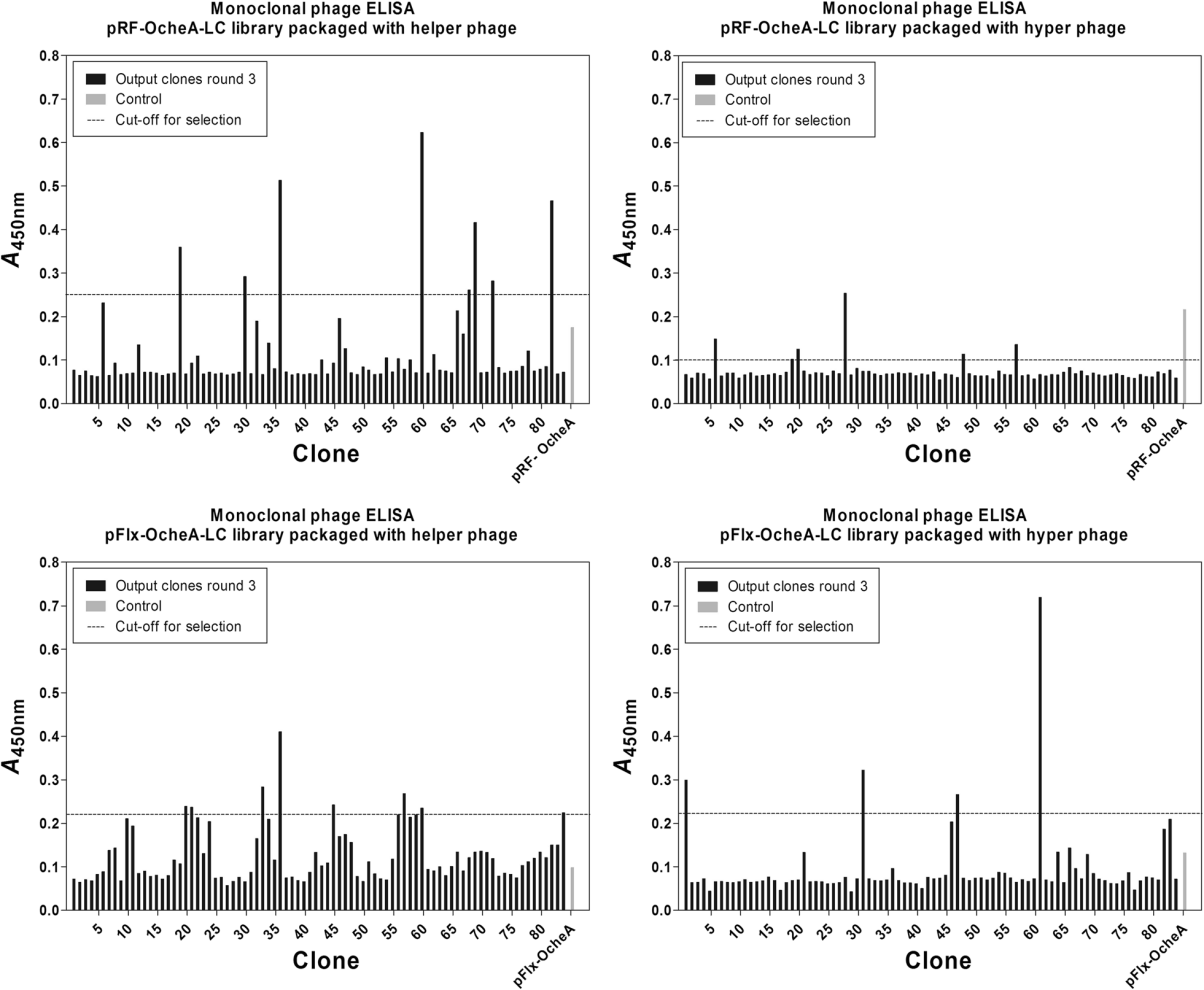
Monoclonal phage ELISAs for the identification of DiCo1-3 specific clones. Of both light chain shuffling libraries, 84 clones from round 3, packaged with helper phages and 84 clones from round 3 packaged with hyper phages were analysed for binding against 500 ng/well of DiCo1-3 mix in a monoclonal phage ELISA. Binding phages were detected with secondary antibody mouse-anti-M13/fd/F1. Clones with values higher than the cut-off (dashed line) were chosen for further binding analysis

We picked From the third selection round, 84 random clones were picked and analysed them by phage ELISA against AMA1 DiCo1-3. The plasmids of the clones with the highest absorptions (above dashed line) were isolated and V gene sequences were analysed (Fig. 5).

Among the selected binders, four different light chain sequences were identified, named LC9, LC16, LC17 and LC17B according to their closest germline V gene identity. Clone 16 was enriched in the pRF-based library using helper phages, and from the pFlx-based library when hyperphages were used. 17B was enriched in the pRF-based library using hyper phages. Clone 9 and 17 were enriched from the pFlx-based library for both packaging phages. All isolated light chains belonged to Vκ1 family and had complementarity determining regions (CDR) of the same lengths (CDR1: 11aa, CDR2: 7aa, CDR3: 9aa according to KABAT definition). Light chain 17 was found to be identical to the parental OcheA light chain with the exception of two residues of the J region, Met-106-Ile and Arg-107-Lys. Light chain 17B differed from light chain OcheA in one point mutation in every CDR and 9 framework residues. Light chain 9 differed from OcheA in 23 residues total of which 8 were CDR residues. Light chain 16 differed most with 25 mutations compared to OcheA total of which 10 were in the CDRs. With germline identities between 93.91% and 97.13% the light chains 9, 16 and 17B were more maturated than the parental chain OcheA (Table 3).

**Table 3:** IMGT/V-QUEST Allele analysis

Light chain	Gene segment	Allele	Score	Nt identity (%)	Identity (nt/nt)	Identity (aa/aa)
9	V-Gene	Homsap IGKV1-9*01 F	1237	93.91	262/279	82/94
	J-Gene	Homsap IGKJ3*01 F	171	97.22	35/36	12/13
16	V-Gene	Homsap IGKV1-16*02 [F]	1264	94.62	264/279	83/94
	J-Gene	Homsap IGKJ5*01 F	167	94.59	35/37	12/13
17	V-Gene	Homsap IGKV1-17*03 F	1372	99.28	277/279	93/94
	J-Gene	Homsap IGKJ2*01 F, or Homsap IGKJ2*02 F	181	97.37	37/38	12/13
17B	V-Gene	Homsap IGKV1-17*03 F	1318	97.13	271/279	90/94
	J-Gene	Homsap IGKJ1*01 F, or Homsap IGKJ4*01 F	144	88.89	32/36	10/13

Germline alleles of V and J-genes were analysed using IMGT/V-QEST for the analysis of human immunoglobulin (IG) or antibody nucleotide sequences. The V- and J-gene alleles with the closest identity to the AMA1-specific light chains are listed. The identity of the phage display derived light chains with their closest germline allele is given as percentage and as number of nucleotides per total nucleotides (nt)

### Expression and characterization of isolated antibody clones

**Fig. 6:**
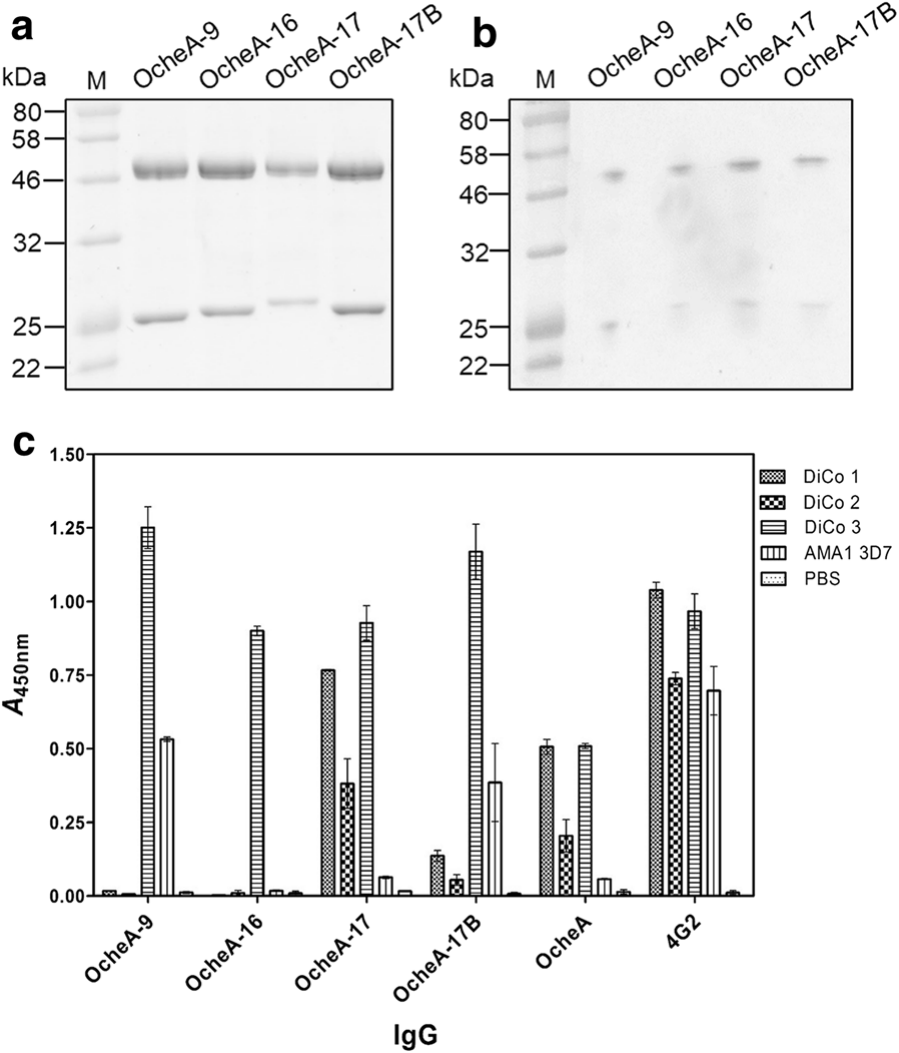
Purity of phage display light chain shuffled antibodies as Immunoglobulin G1 and indirect ELISA against AMA1-variants. Antibodies were transiently expressed as human IgG1/κ in *N. benthamiana* and purified using Protein A-chromatography. **a** SDS-PAGE Coomassie Brilliant blue stained, **b** Western blot, anti-human-IgG (H+L)-AP (1:5000) + NBT/BCIP. Dividing lines in B indicate parts from the same blot that were re-arranged. **c** Indirect ELISA against AMA1-variants with plant-expressed human IgG1/κ. The four selected light chain-shuffled antibodies OcheA-9, OcheA-16, OcheA-17 and OcheA-17B, parental antibody OcheA and AMA1-specific antibodies 1E4 and 4G2 were analysed in the presence (100 ng/well) and absence (PBS) of the antigens DiCo1-3 and AMA1-GKO (glyco-knockout, strain 3D7). Values represent mean ± SD of triplicate measurements after subtraction of the secondary antibody-control values

The four different V gene sequences were cloned into the plant expression vector pTRAkt and transiently expressed as full-size human IgG1/κ immunoglobulins in *N. benthamiana*. Expression levels increased 20 to 80-fold compared to the parental clone (110–440 mg/ kg FLW). Antibodies were purified by protein A affinity chromatography. The purity (95%) of the antibodies was confirmed by SDS-PAGE followed by staining with Coomassie Brilliant Blue (Fig. 6a), and by western blot analysis followed by detection of the antibody heavy and light chains using mouse-anti-human IgG(H+L) antibodies conjugated to AP (Fig. 6b). The heavy and light chains migrated at their predicted sizes of ~ 50 kDa and ~ 25 kDa, respectively.

To confirm specific binding of the purified full-size antibodies the binding activity was studied by ELISA and SPR spectroscopy. Indirect ELISA was used to determine whether the four antibodies bound to AMA1 Dico1–3 and an AMA1 3D7 glyco-knockout variant. Microtiter plates were coated with the antigens (50 ng/well) and the protein A-purified antibodies were applied (200 ng/well). As control, AMA1-antibody 4G2 was applied. In this experiment, all antibodies recognized AMA1 DiCo3. The signals were 1.7 to 2.3-fold higher for the shuffled antibodies as compared to the parental antibody OcheA and they were also similar or higher than for control antibody 4G2. Furthermore, antibodies 17 and 17B also showed some binding activity against AMA1 DiCo1, DiCo2 and AMA1 3D7, which was considerably less than the respective binding activity of the control antibody 4G2 (Fig. 6c).

In this ELISA, all four antibodies bound at least to AMA1 variant DiCo3, and the analysis of binding affinity by SPR spectroscopy confirmed that all four antibodies had the greatest affinity to AMA1 DiCo3 (OcheA17B had the lowest affinity constant of ~ 1.7 × 10^–9^ M). The specific binding affinity of the antibodies OcheA-17B to AMA1 3D7 was weaker but detectable (176 nM, Table 4), the affinity of the other antibodies to AMA1 3D7 could not be determined.

**Table 4:** Expression levels and affinity constants assessed by SPR spectroscopy

	OcheA-9	OcheA-16	OcheA-17	OcheA-17B
Expression level (mg/kg FLM)	150	140	440	110
AMA1 DiCo1 K_D_ (nM)	–	–	213	43
AMA1 DiCo2 K_D_ (nM)	110	–	160	1750
AMA1 DiCo3 K_D_ (nM)	68	55	61	1.7
*Pf*3D7 AMA1 K_D_ (nM)	–	–	–	176

To calculate expression levels the concentrations of recombinant antibodies OcheA-9, OcheA-16, OcheA-17 and OcheA-17B in *N. benthamiana* plant extracts were assessed measuring the capture level on a protein A surface in relation to an internal antibody standard using BiacoreT200. For affinity kinetic measurements purified recombinant antibodies OcheA, OcheA-9, OcheA-16, OcheA-17 and OcheA-17B were captured on a protein A surface (110, 110, 130, 140, 120 RU respectively) and the antigens were injected successively in concentrations of 360 nM, 180 nM and 90 nM for 180 s (30 µL/min). The surface was washed with HBS-EP buffer and regenerated with 30 mM HCl between measurements. Data were evaluated using T200EvaluationSoftware3.0 using the two state reaction binding model. “–”: not determinable

**Fig. 7:**
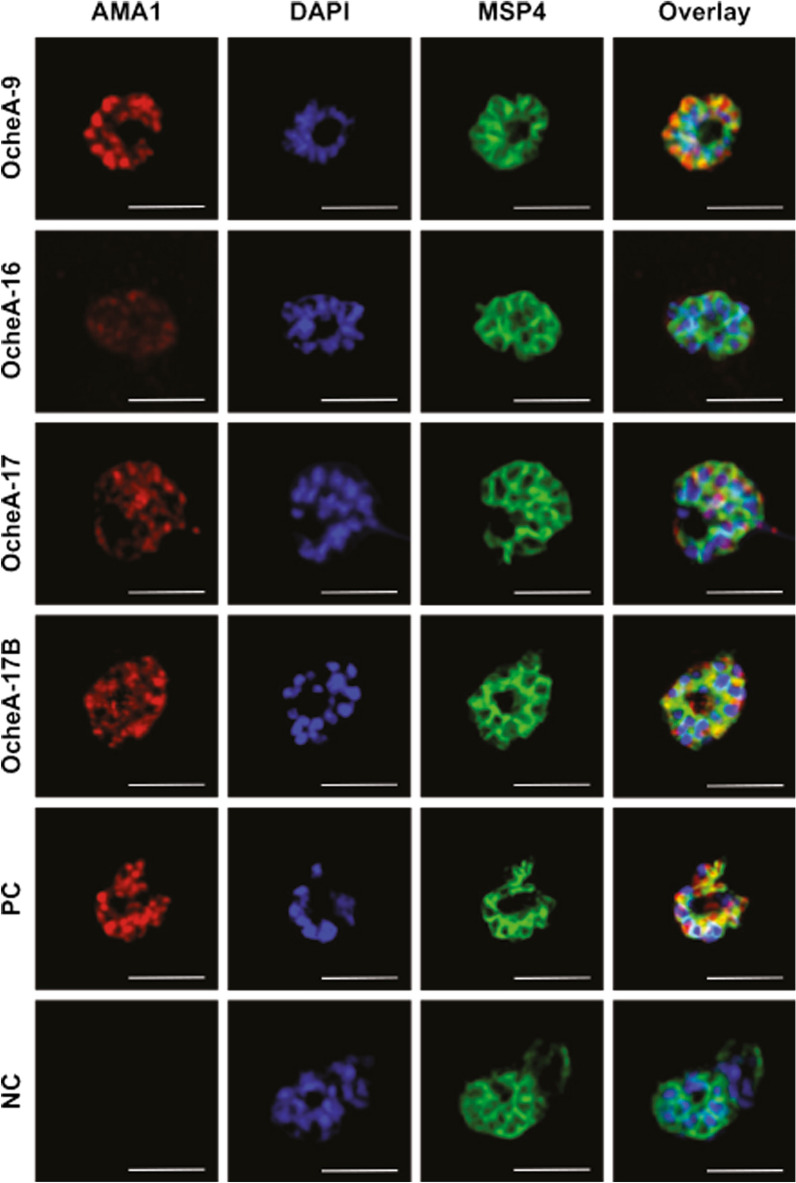
Immunofluorescence assay with methanol fixed schizonts of strain 3D7. Nuclei were stained with DAPI (blue). Co-staining with mouse-anti-MSP4 antibody 2.44, detected by goat-anti-mouse-IgG (H+L)-Alexa488 [14]. AMA1 antibodies were applied at 50 µg/mL and detected with goat-anti-human IgG (H+L)-Cy3 [59]. Scale bar: 5 µm

In an immunofluorescence assay (IFA) using *P. falciparum* strain 3D7, binding of the antibodies to the apical end of the parasites was detected on schizonts by all four isolated antibodies, whereas for OcheA-9 and OcheA-17B the AMA1 (Cy3) signal intensity was comparable to the polyclonal AMA1-specific positive control antibody BG98 suggesting binding to native, membrane-bound AMA1 is specifically recognized. There was no Cy3 signal from the negative control. (Fig. 7).

### Functional activity of selected recombinant humAbs

**Fig. 8:**
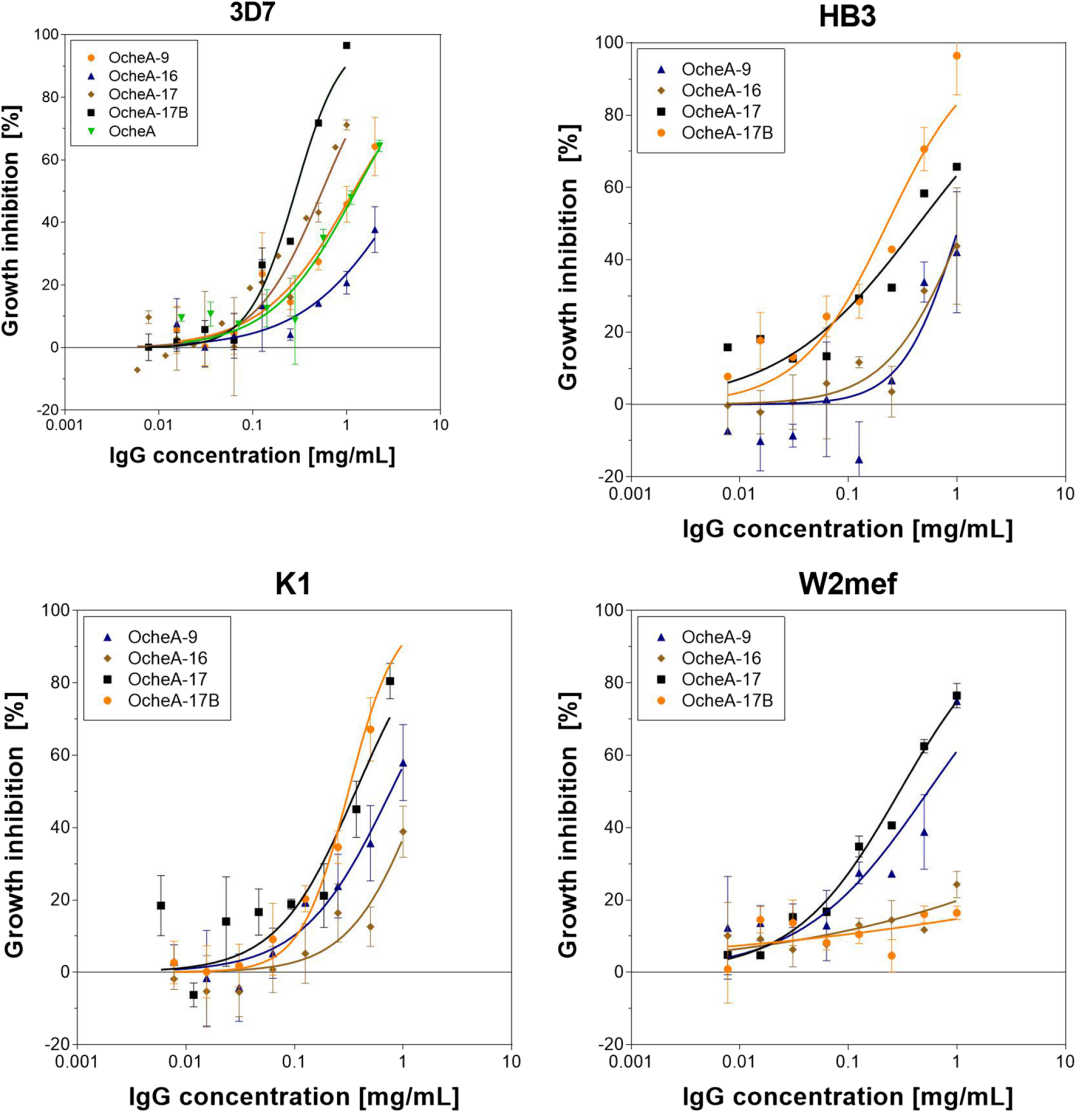
Growth inhibition of light chain shuffled antibodies OcheA-9, OcheA-16, OcheA-17 and OcheA-17B. Growth inhibitory activity was assessed using growth inhibition assay. Synchronized schizonts were incubated at a parasitaemia of 0.3% with the antibodies. After 48 h parasites were harvested, the pLDH activity quantified and the growth inhibition calculated. Error bars represent mean ± SEM values for two to three repeated assays (n = 2–3) carried out in triplicates. Data were analysed using the "log (agonist) vs. response—find ECanything" function of GraphPad Prism

Because all shuffled recombinant antibodies specifically bound AMA1 in ELISA, SPR and IFA, the functionality of the antibodies was evaluated in vitro using growth inhibition assays. The antibodies were tested at different concentrations starting at 1 mg/mL or 2 mg/mL against strains 3D7, K1, HB3 and W2mef. All antibodies inhibited the merozoite invasion in strains 3D7, K1 and HB3 at the applied concentrations to varying extent, and the mefloquine-resistant strain W2mef was also inhibited. The inhibitory activity of the different antibodies was concentration-dependent and strain-dependent. The lowest EC_50_ values (0.2–0.3 mg/mL) were achieved by antibody 17B against strains 3D7, HB3 and K1, and the broadest inhibitory activity was achieved by antibody 17, with an EC_50_ of less than 0.3–0.55 mg/mL for all four tested strains (Fig. 8 and Table 5).

**Table 5:** Inhibitory concentrations of the affinity maturated antibodies against the *P. falciparum* strains 3D7, HB3, K1 and W2mef

*P. falciparum* strain	EC_50_ (95%CI) (mg/mL)			
	Antibody			
	OcheA-9	OcheA-16	OcheA-17	OcheA-17B
3D7	1.19	> 2	0.55	0.29
	(0.79–1.79)		(0.43–0.70)	(0.24–0.34)
HB3	1.07	1.18	0.45	0.23
	(0.61–1.86)	(0.65–2.13)	(0.25–0.81)	(0.17–0.32)
K1	0.77	1.50	0.37	0.31
	(0.49–1.21)	(0.79–2.87)	(0.24–0.55)	(0.26–0.38)
W2mef	0.55	> 2	0.30	> 2
	(0.31–0.96)		(0.26–0.34)	

### Affinity and pairwise epitope mapping of recombinant humAbs

**Fig. 9:**
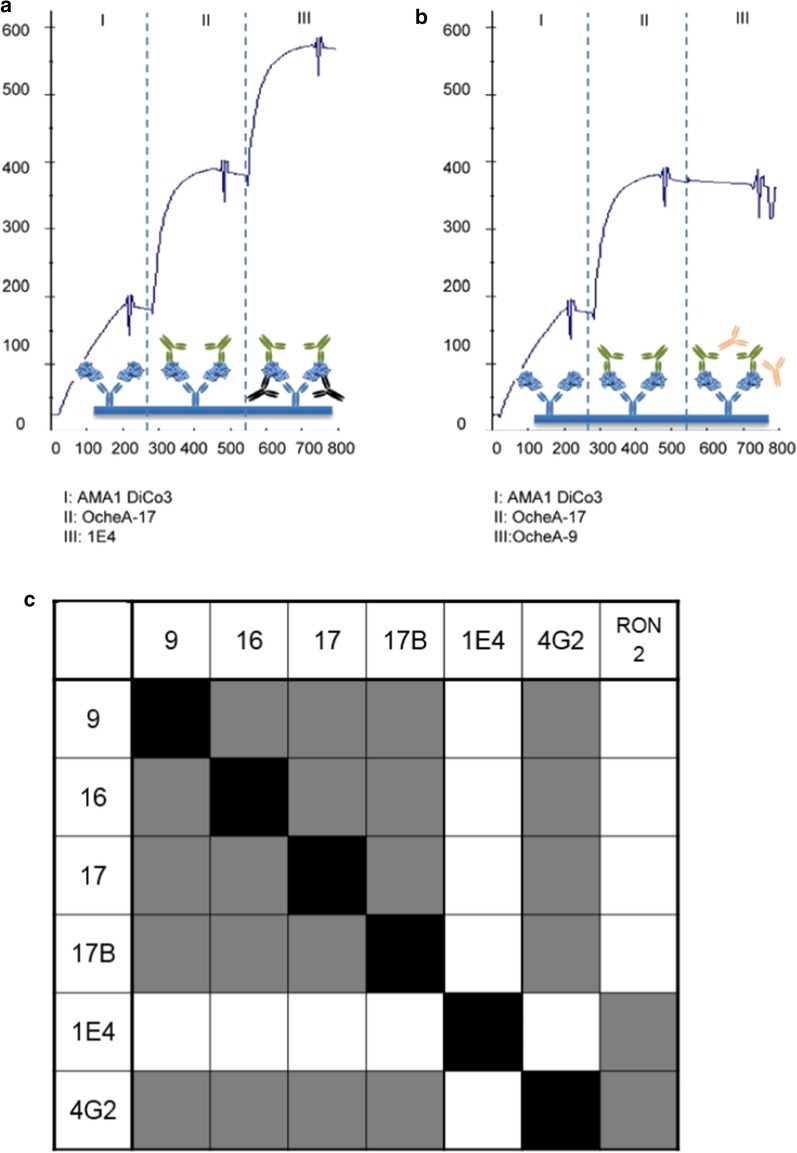
Pairwise epitope mapping using SPR spectroscopy. For competition measurements of the antibodies generated by phage display among themselves as well as to other described antibodies, the AMA1-antigen DiCo3 was captured on a surface coated with anti-AMA1 antibody 1D7. First the saturating antibody was injected (180 s, 30 µL/min). If there was no binding of the subsequently injected competing antibody, binding competition was assumed. Antibodies were tested against all other available AMA1-antibodies pairwise. Represented are exemplary curves for a non-competing antibody pair (OcheA-17/1E4) (**a**) and a competing (OcheA-9/OcheA-17) antibody pair (**b**). The results of all pairwise epitope mappings being performed is summarized (**c**)

The varying affinities of the antibodies to different AMA1 variants and the different inhibitory activities against different *P. falciparum* strains could be explained by polymorphisms in the epitope sequence or epitope drift. Therefore, we sought to map the approximate epitopes by SPR-based competition assays. The four shuffled antibodies were combined in pairs and competition was measured in the presence of AMA1 DiCo3, because all four antibodies bound with relatively high affinity to this AMA1 variant. Furthermore, competition with the antibodies 1D7, 1F9, 4G2 and 1E4 was investigated in the presence of AMA1 3D7. No competition could be detected between the isolated antibodies and the human anti-AMA1 antibody clone 1E4, binding to AMA1 domain I [58]. All four anti-AMA1 isolated by phage display antibodies competed with each other in the presence of AMA1 DiCo3 and also with 4G2 in the presence of AMA1 3D7. These data suggested that the shuffled antibodies bind to epitopes that either overlap with the epitope sequence of 4G2 or inhibit its binding by steric hindrance. Exemplary SPR competition results as well as a summary Table presented in Fig. 9.

A similar approach was used to investigate whether the antibodies compete with RON2 for binding, a mechanism which has been hypothesized for the mode of inhibition of anti-AMA1 antibodies. SPR analysis revealed that there is binding of the AMA1 antibodies to AMA1 pre-incubated with (and, therefore, in complex) with RON2 suggesting that there is no competition with RON2, while anti-AMA1 antibody 4G2 did not bind the AMA1-RON2 complex in this experiment indicating competition with RON2 for AMA1 binding (Fig. 9c).

## Discussion

Here, we describe the isolation of an AMA1-specific human monoclonal antibody by phage display and its significant improvement of its affinity and expression by light chain shuffling. The quality of a library depends on several factors like B cell source (donor selection), library size and library diversity.

The choice of the B-cell source was based on the selection of donors with proven reactivity against a high variety of malaria antigens, including high reactivity against AMA1 [46, 50, 58, 60, 61]. The immune library had a final size of 1.9 × 10^7^ clones and featured sequences from most VH and Vκ families. The most represented families were VH1, VH3, Vκ1 and Vκ4. This differed from the reported predominance of the VH3 family in the normal human repertoire. The VH2, 5, and 6 in the normal human repertoire are used at much lower frequencies [62]. The VH4 family, in comparison to the normal human repertoire, was underrepresented in this library, however the cloning strategy did not aim at reproducing the in vivo V gene distribution in detail. For the Vκ families, except for Vκ5, all the other families were represented. This is reflective of the present knowledge about the predominance of Vκ families 1–4 in the normal human repertoire [63]. The absence of the Vκ5 family could be due to the generally low frequency of usage or the small number of clones sequenced.

After four rounds of panning, one dominant clone and five other AMA1-specific clones were enriched as analysed by monoclonal phage ELISA. The two clones with the highest absorption signals were transiently expressed as human IgG1/κ antibodies in *N. benthamiana.* One of the antibodies retained strong binding activity, and although it was expressed at a low level (6 mg/kg fresh leaf weight) its ability to bind AMA1 was confirmed by SPR spectroscopy and it was also shown to inhibit the growth of *Pf* strain 3D7 although with comparably low efficiency.

A low affinity is typical for initial antibodies generated by phage display and affinity maturation is often necessary. Several methods have been described, including random mutagenesis of CDRH3 and chain shuffling, resulting in the generation of affinity-matured variants [43, 64]. When the antibody sequence was compared to germline sequences using the IMGT/V-Quest analysis tool [47], we observed 99.22% identity to allele IGκV1-17*03, whereas the heavy chain showed only 83.33% identity to IGHV1-69*11, suggesting a higher degree of maturation.

In an attempt to increase the affinity and perhaps achieve a native match, light chain shuffling was used. It is known that heavy chains can be promiscuous allowing the isolation of more than one matching light chain while retaining antigen specificity [65]. VH1-69 is a frequently used VH segment [66–69].

The light chain repertoire was constructed from the same cDNAs as the initial combinatorial immune library. The repertoire was cloned in vector pRFII containing the heavy chain of the isolated clone and was subcloned in pFlx so that both libraries could be panned in parallel using two different packaging phages for every library. Vector pFlx was efficient for the panning of Fabs and in was more stable than pRF with a higher percentage of clones with complete inserts among the output clones as compared to the pRF based library. The use of different packaging phages led to the enrichment of different clones. Rondot et al. who generated the “hyperphages” observed increased output titers and enrichment factors for these phages compared to helper phages [70]. This was not the case for our panning experiments. All phage yields of the final panning rounds were in the same range regardless of the packaging phage used, however the phage yields after round 1 and two were higher when hyperphage were used. The monoclonal phage ELISA showed that the fraction of binders in the third panning round was larger for helper phage pannings, but the hyperphage method led to the isolation of the binder with the highest affinity (antibody 17B from library pRF). This is unexpected since the increase of avidity when using hyper phage should theoretically allow for the isolation of lower affinity binders and thereby increase the total number of enriched clones.

All four enriched light chain sequences were similar to the parental light chain, in agreement with previous chain shuffling results [71]. This matches the observation that only selected and often similar light chains can pair with a given heavy chain [72–74]. The binder with the highest affinity had the same germline identity (IMGT/V-Quest) as the parental light chain (IGKV1-17*03), but there was one amino acid substitution in every CDR. The parental kappa chain, and both sequences 17 and 17B, possibly arose from the same initial B-cell clone by somatic hypermutation, representing different maturation stages of a gene isolated from the same donor. It is unlikely that the mutations that distinguish 17B from the parental light chain and 17 were introduced by PCR, because they are concentrated in the CDRs. Light chain 17 is identical to the parental light chain with the exception of the two final residues in the variable region. As expected, the mutations Met-106-Ile and Arg-107-Lys in the J region did not have an impact on affinity. However, the mutations came along with an increase of the expression level of the antibody of ~ 73-fold compared to the parental antibody, maybe due to an impact on improved folding or stability of the antibody. The increased expression level was a case of serendipity, because obviously expression yield is not a selection criterium of phage display. Chain shuffling can be used however, to select for antibodies with improved prokaryotic expression levels.

All clones were confirmed to be specific to AMA1 by ELISA and SPR spectroscopy but there were differences in specificity towards certain AMA1 variants. In conclusion, all four antibodies had overlapping epitopes because they all competed with each other and with mAb 4G2. This would imply that no major epitope drift occurred when the light chain was replaced, although an epitope drift after chain shuffling did occur. Differences in specificity thus most likely result from AMA1 polymorphisms in the epitope. This hypothesis could be investigated in the future by crystallization or other methods like linear or conformational epitope mapping as used for the fine epitope mapping of 1E4, 4G2 and 1F9 [54, 32, 58].

The detailed investigation of the mAbs 1E4, 1F9 and 4G2 identified epitopes of natural inhibitory antibodies within AMA1. Antibodies 1E4, 1F9 and 4G2 have been shown to bind to residues in domains I and II between which AMA1 forms a hydrophobic trough. While the binding of 1F9 and to a lesser extend 1E4 depends on a highly polymorphic residue in domain I in an extremely polymorphic region attributed to immune evasion, 4G2 was shown to bind to residues in domain II which are greatly conserved among plasmodial AMA1, thus rendering mAb 4G2 pan-specific and more broadly inhibitory. Because all four antibodies competed with mAb 4G2 it is possible that they bind in this conserved region, which would also explain their ability to inhibit all five tested *P. falciparum* strains. Single polymorphisms in this conserved region may contribute to the differences observed in affinity and inhibition. Competition of the AMA1 ligand RON2 with mAb 4G2 has been reported. RON2, which is thought to bind close to the hydrophobic trough, is necessary for the formation of the moving junction complex for the invasion of red blood cells, a process which is still not completely understood. The antibodies described here do not compete with RON2 for AMA1 binding and seem to inhibit via a different mode of action.

Indeed, all shuffled antibodies were able to inhibit the growth of *P. falciparum* strains 3D7, HB3, K1, and W2mef. The lowest EC_50_ values were observed for clone 17B against strains 3D7, HB3 and K1, the clone with the highest affinity to AMA1 DiCo3. For the same antibody, inhibitory activity could not be confirmed at a concentration of 1 mg/mL against strain W2mef. Only antibody OcheA-17 achieved EC_50_ values below 1 mg/ mL against all five tested strains, while its affinity for AMA1 3D7 could not be determined and increase in affinity to DiCo3 was moderate (K_D_ ~ 6 × 10^–8^ against AMA1 DiCo3). It inhibited the corresponding strain 3D7 with an EC_50_ of 0.25 mg/mL. Previously, the inhibitory capacity of the antibody humAbAMA1 (clone 1E4) in comparison to the murine and rat antibodies 1F9 and 4G2 was demonstrated. The antibody humAbAMA1 showed high activity against the strain 3D7 (EC50: 0.035 mg/mL), but much lower activity against other strains (3 to 6 times higher EC50) [58]. The strain dependency of the inhibitory activities of these antibodies is highly interesting and stresses the importance of antibody epitopes. As the novel antibodies described here partially have to strain-dependency of their activity, the relevant epitopes might be. In future work, it would be highly interesting to investigate the synergistic inhibitory effects of the antibodies recognizing different epitopes on the same antigen.

Despite an identical light chain V region with parental clone OcheA, the inhibitory activity of OcheA-17 was higher than that of OcheA. This implies that not only the low expression level but also the approximately fivefold lower inhibitory activity results from two mutations in the light chain J region of OcheA compared to OcheA-17. Although the J region sequence of OcheA can be found in vivo according to the IMGT database, this amino acid sequence might be problematic for *N. benthamiana* expression and thus result in misfolded or unpaired antibody molecules impairing their functionality.

Assuming no epitope drift occurred due to the light chain shuffling, the results for affinity of the four antibodies against 3D7 and inhibitory activity against the corresponding parasite strains imply that the affinity constant is not necessarily a measure for the inhibitory activity of an AMA1-specific antibody. More experiments are needed to confirm this or identify other factors that decrease the EC_50_ of an inhibitory antibody. A low EC_50_ is required for the therapeutic use of antibodies at low doses for reducing production costs. Improving the cross-strain specific inhibitory activity of monoclonal antibodies is an important step towards the generation of an antimalarial antibody therapy. The EC_50_ values obtained here are in a range that are comparable to other anti-merozoite antibodies. In combination with cellular immune responses by e.g. monocytes or neutrophils, the anti-malarial activity of the antibodies might even be increased in vivo and the necessary therapeutic dose reduced accordingly [75].

When scoring the sera of the donors from the study population, a significant correlation was found between the presence of AMA1-specific antibodies and inhibition capacity [46]. The heavy chain which caused the binding against AMA1 was isolated from an immune library constructed from the V genes of these donors and despite the possibility that the native chain pairing is not among the chain shuffled antibodies, the antibody sequences were maturated in response to natural malaria infections and might be contributing to the protective malaria immunity in the respective patients. The observation that functionally active clones, homologous or identical to OcheA were isolated during the chain shuffling, suggests that one or several maturation stages of an OcheA light chain homologue might be the in vivo match of the OcheA heavy chain. A compact immune human phage display library is sufficient for the isolation of potent inhibitory monoclonal antibodies.

This is the first report describing the isolation of a human monoclonal antibody against AMA1 from a human immune antibody phage display library. The generated library or comparable libraries can be used to isolate human monoclonal antibodies against a variety of *P. falciparum* antigens.

One limitation of the study might be the number of isolated antibodies. Nevertheless, applying the described process to a pre-characterized human sample set generates a library with broad potential, but chances of reproducing the native heavy/light chain pairing from a original B-cell are strongly dependent from the total number of reactive B cells being isolated from a patient/patient population and might be comparably low. Nevertheless, other technologies, like sorting of single B-cells, culturing B-cells at very low cell numbers, or sequencing B-cells using single-cell droplet microfluidics are more suitable to isolate antibodies with native chain pairing [48, 58, 76]. The phage display method described here is useful for improving antibodies that originate from such original pairings. Chain shuffling or random mutagenesis of Fab-sequences and subsequent phage display methodology could generate antibodies with better binding capacity and functionality. Improvements of the technology might include (a) increasing the size of the library depending on the number of reactive B cells accessible from patients, (b) including of both light chain types, lambda and kappa, (c) pre-selection of antigen-specific B cells prior to the construction of the library (e.g. by antigen-specific B-cell sorting) and (d) changing the cloning strategy to have native pairing of the heavy and light chain. A subsequent heavy chain shuffling could also further improve affinity of the antibodies.

The coincidental finding that a minor sequence mutation substantially increased the expression level in *N. benthamiana* suggests that chain shuffling could also be beneficial for this very purpose. An increase of expression levels in a different expression platform than *E. coli* however is nothing that can be selected for during the panning. Therefore, the success depends on the promiscuity of the respective antibody chain, because a panel of shuffled antibodies would be desired. Furthermore, the generation of more specific shuffled variants increases the chance to find clones with increased expression levels or other improved properties. The shuffled clones already had comparably high expression levels in *N. benthamiana* (100–440 mg/kg FLM) which could be further improved in the 1–3 g/kg FLM range. The production of antibodies in plants such as tobacco is an economic alternative that may be suitable for developing-country applications [77]. The antibody cocktail ZMapp, which was used for the treatment of Ebola patients during the 2014 outbreak in West Africa was produced by transient expression in *N. benthamiana* as well suggesting that this platform may also be suitable for the rapid, inexpensive and scalable production of therapeutic malaria antibodies [78]. Because the generated AMA1-antibodies have comparably high expression levels and a cross-strain specific inhibitory activity against *P. falciparum*, they might be used as a basis for the development of an antibody cocktail for future prophylactic or therapeutic anti-malarial strategies. In any case, they are valuable tools for the study of AMA1 dependent invasion inhibition of *Plasmodium falciparum* and the correlation of epitope, affinity and growth inhibitory activity, ultimately contributing important knowledge for the rational design of next-generation malaria vaccines.

## Supplementary Information

**Additional file 1: Table S1.** Kappa V gene amplification and reamplification primers. **Table S2.** Cloning primers (pTRAkt).
