# Supplementary material for: Isolation and light chain shuffling of a Plasmodium falciparum AMA1-specific human monoclonal antibody with growth inhibitory activity
Source: Malar J. 2021 Jan 11;20:37. doi: 10.1186/s12936-020-03548-3 (PMC7798374; doi:10.1186/s12936-020-03548-3)
Supplement: Supplementary file 1 — Additional file 1: Table S1. Kappa V gene amplification and reamplification primers. Table S2. Cloning primers (pTRAkt). [file 12936_2020_3548_MOESM1_ESM.docx]

**Additional Tables**

Table S1. Kappa V gene amplification and reamplification primers

| Primer | Nucleotide sequence (5‘→3‘) |
| --- | --- |
| Vκ1a_Vbase | RACATCCAGATGACCCAG |
| Vκ1b_Vbase | GMCATCCAGTTGACCCAG |
| Vκ1c_Vbase | GCCATCCRGATGACCCAG |
| Vκ1d_Vbase | GTCATCTGGATGACCCAG |
| Vκ2a | GATATTGTGATGACCCAGACTCC |
| Vκ2b_Vbase | GATRTTGTGATGACTCAG |
| Vκ3a | GAAATTGTGCTGACACAGTCTCC |
| Vκ3a_Vbase | GAAATTGTGTTGACRCAG |
| Vκ3b | GAAATTGTGCTGACTCAGTCTCC |
| Vκ3b_Vbase | GAAATAGTGATGACGCAG |
| Vκ3c_Vbase | GAAATTGTAATGACACAG |
| Vκ3d | GAAATTGTGTTGACTCAGTCTCC |
| Vκ4 | GACATCGTGATGACCCAGTCTCC |
| Vκ5 | GAAACGACACTCACGCAGTCTCC |
| Vκ6b_Vbase | GATGTTGTGATGACACAG |
| Vκ8 | GAGATTGTGATGACCCAGACTCC |
| Vκ10 | GACCACGTGATGACCCAGTCTCC |
| Cκ-new | ACACTCTCCCCTGTTGAAGCTCTT |
| Vκ1a*Apa*LI_Vbase | TGAGCACACAGTGCACRACATCCAGATGACCCAG |
| Vκ1b*Apa*LI_Vbase | TGAGCACACAGTGCACGMCATCCAGTTGACCCAG |
| Vκ1c*Apa*LI_Vbase | TGAGCACACAGTGCACGCCATCCRGATGACCCAG |
| Vκ1d*Apa*LI_Vbase | TGAGCACACAGTGCACGTCATCTGGATGACCCAG |
| Vκ2a (*Apa*L1) | TGAGCACACAGTGCACGATATTGTGATGACCCAGACTCC |
| Vκ2b*Apa*LI_Vbase | TGAGCACACAGTGCACGATRTTGTGATGACTCAG |
| Vκ3a (*Apa*L1) | TGAGCACACAGTGCACGAAATTGTGCTGACACAGTCTCC |
| Vκ3a*Apa*LI_Vbase | TGAGCACACAGTGCACGAAATTGTGTTGACRCAG |
| Vκ3b (*Apa*L1) | TGAGCACACAGTGCACGAAATTGTGCTGACTCAGTCTCC |
| Vκ3b*Apa*LI_Vbase | TGAGCACACAGTGCACGAAATAGTGATGACGCAG |
| Vκ3c*Apa*LI_Vbase | TGAGCACACAGTGCACGAAATTGTAATGACACAG |
| Vκ3d (*Apa*L1) | TGAGCACACAGTGCACGAAATTGTGTTGACTCAGTCTCC |
| Vκ4 (*Apa*L1) | TGAGCACACAGTGCACGACATCGTGATGACCCAGTCTCC |
| Vκ5 (*Apa*L1) | TGAGCACACAGTGCACGAAACGACACTCACGCAGTCTCC |
| Vκ6b*Apa*LI_Vbase | TGAGCACACAGTGCACGATGTTGTGATGACACAG |
| Vκ8 (*Apa*L1) | TGAGCACACAGTGCACGAGATTGTGATGACCCAGACTCC |
| Vκ10 (*Apa*L1) | TGAGCACACAGTGCACGACCACGTGATGACCCAGTCTCC |
| Cκ-new-*Not*I | GAGTCATTCTCGACTTGCGGCCGCACACTCTCCCCTGTTGAAGCTCTT |

**Table S2. Cloning primers (pTRAkt)**

| Primer | Nucleotide sequence (5’→3’) | Purpose |
| --- | --- | --- |
| VHforAge | AAAAAAAAACCGGTCAGGTCCAGCTTGTGCAGTCTGGG | OcheAmut (forward) |
| VHrevSal | AAAAAAAAGTCGACGCTGAGGAGACGGTGACCAGGGTTCC | OcheAmut (reverse) |
| 5’AgeI Vκ1-9 mut | TTGTGCTGCAACCGGTGTACATTCCGCCATCCAGTTGACCCAGTCT | LC9 (forward) |
| BsiWI Jκ3 | GCCACCGTACGTTTGATATCCACTTTGGTC | LC9 (reverse) |
| 5’AgeI Vκ1-16 | CTGCAACCGGTGTACATTGTGCCATCCAGTTGACCCAGTC | LC16 (forward) |
| 5’BsiWI Jκ 6 | GCCACCGTACGTTTAATATCCAGTCGTGTC | LC16 (reverse) |
| 5’AgeIVκ1-17 | CTGCAACCGGTGTACATTGTGACATCCAGATGACCCAGTC | LC17 (forward) |
| 3’BsiWI Jκ2 | GCCACCGTACGTTTGATCTCCAGCTTGGTC | LC17 (reverse) |
| 5’Age Vk17-02 OcheAhyper28 | CTGCAACCGGTGTACATGGGAGAATTGTGATGACCCAGAC | LC17B (forward) |
| BsiWI Jκ1/4 OcheAhyper28 | GCCACCGTACGTTTGACTTCCACCTTGGTC | LC17B (reverse) |
